# Machine Learning-Powered Vision for Robotic Inspection in Manufacturing: A Review

**DOI:** 10.3390/s26030788

**Published:** 2026-01-24

**Authors:** David Yevgeniy Patrashko, Vladimir Gurau

**Affiliations:** Robotics Process Development Laboratory (RPDL), Georgia Southern University, Statesboro, GA 30458, USA; dp16651@georgiasouthern.edu

**Keywords:** machine learning in manufacturing, deep learning in robotic vision inspection, computer vision for industrial quality control

## Abstract

Machine learning (ML)-powered vision for robotic inspection has accelerated with smart manufacturing, enabling automated defect detection and classification and real-time process optimization. This review provides insight into the current landscape and state-of-the-art practices in smart manufacturing quality control (QC). More than 50 studies spanning across automotive, aerospace, assembly, and general manufacturing sectors demonstrate that ML-powered vision is technically viable for robotic inspection in manufacturing. The accuracy of defect detection and classification frequently exceeds 95%, with some vision systems achieving 98–100% accuracy in controlled environments. The vision systems use predominantly self-designed convolutional neural network (CNN) architectures, YOLO variants, or traditional ML vision models. However, 77% of implementations remain at the prototype or pilot scale, revealing systematic deployment barriers. A discussion is provided to address the specifics of the vision systems and the challenges that these technologies continue to face. Finally, recommendations for future directions in ML-powered vision for robotic inspection in manufacturing are provided.

## 1. Introduction

Smart manufacturing uses real-time data and data-driven technologies such as artificial intelligence (AI), cloud connectivity and industrial internet of things (IIOT) to increase the efficiency and agility of traditional manufacturing systems. It uses data from sensors, machines, and across the supply chain to improve quality, optimize production and to respond in real time to changing demands and conditions in the factory, supply network, and customer needs. Manufacturers are under pressure to rapidly adapt, and many are turning to smart manufacturing technologies to address challenges in labor shortages, skills gaps, and geopolitical and supply chain issues.

The current interest in smart manufacturing is reflected in the 2025 State of Manufacturing Report conducted by Rockwell Automation [1]. They analyzed 1560 questionnaires sent to decision-makers in manufacturing industries around the world. A total of 95% of the responders reported that they have either invested in, or plan to invest in, Machine Learning (ML), GenAI or Causal AI in manufacturing in the next five years. Among respondents, 50% plan to use AI/ML in quality control (QC), 49% in cybersecurity, 42% in process optimization, 37% in robotics, and 36% in logistics.

Traditional machine vision (MV), or rule-based MV has been, for decades, an essential tool in manufacturing, facilitating QC tasks such as gaging, defect detection, sorting parts, or assembly verification through the detection and localization of parts. To achieve these tasks, industry-level MV uses techniques such as edge detection, template matching, color analysis, morphological operations, or stereo imaging. Traditional MV provides techniques for camera calibration [2,3] used to correct lens distortions and to convert image pixels to real world coordinates. In 3D vision applications, MV provides techniques for stereo calibration [4,5] used to find the intrinsic parameters for each of the two cameras and the extrinsic parameters between the two cameras. When MV is used with robotic technology, it provides techniques for Hand–Eye calibration [6,7,8,9,10], or Robot–World–Hand–Hand–Eye calibration [11,12], used to determine the position and orientation of the coordinate system associated with the camera sensor relative to the coordinate system associated with the robot tool center point, and that of the target object relative to the camera sensor. These latter two techniques enable the camera to guide the robot in its work envelope and execute tasks.

Traditional MV works well for tasks with limited variability but fails to meet expectations when handling large product variations or unpredictable defects. Nevertheless, recent advancements in AI have led to the emergence of a new approach: deep learning (DL)-enhanced MV, which offers greater flexibility and adaptability in real-world applications. Unlike traditional MV, DL-enhanced MV significantly improves its accuracy in applications with large product variations, applications with unpredictable defects, or in complex environments.

The integration of DL-enhanced MV with robotics has significantly boosted inspection capabilities even further. Unlike fixed-camera inspection systems, vision-guided robotics, also known as “eye-in-hand” systems, can dynamically adapt the inspection path around objects to navigate through confined spaces or to scan along irregular features. This flexibility further improves the QC efficiency in systems with large positional, dimensional, or visual variability. In these cases, the integrated ML algorithms have a two-fold beneficial impact on the efficiency of the QC operation: they enhance the detection capabilities of the vision system, and at the same time, provide the robot with the ability to perceive and understand its environment, allowing it to adapt in real time to changes and variations in the production line. The interested reader may find additional information on the ML-enhanced vision-based control of robots in manufacturing in [13,14,15,16,17,18,19,20,21].

The objective of this literature review is to obtain information on the current landscape and the state-of-the-art practices in smart manufacturing QC and to extract details of ML-enhanced vision for robotic inspection, regarding the following:

- The manufacturing context, such as the industry sector or application domain, production environment characteristics (high-mix/low-volume, assembly line, etc.), integration with existing systems (Industry 4.0, IoT, collaborative robots), operational constraints or requirements, or the scale of implementation (prototype, pilot, full deployment).
- The system implementation, such as the robot integration approach, the vision system used, camera type and specifications (2D, RGB-D, stereo), additional sensors used (structural light), data fusion approaches, etc.
- The ML approaches used, such as specific algorithms, training methodology, data preprocessing and augmentation techniques, feature extraction methods, or model architecture.
- Performance metrics, such as accuracy for detection/classification, false positives/negatives if reported, detected rates, comparison with baseline, processing speed or inference time, etc.

## 2. Review Approach

A first semantic search was performed, assisted by Elicit search engine, of 138 million academic papers using the following query: “Machine learning-powered vision for robotic inspection in manufacturing”. The query retrieved 500 of the most relevant papers, which were screened to meet the following criteria:

- Does the study involve robotic systems equipped with computer vision capabilities for inspection tasks?
- Is the application specifically within manufacturing environments such as production lines, quality control, or assembly inspection?
- Does the study explicitly incorporate ML algorithms for vision processing such as DL, neural networks (NN), or traditional ML approaches, rather than being purely rule-based or using only traditional image processing?
- Does the research focus on inspection, quality control, defect detection, or monitoring applications?
- Does the study report quantitative or qualitative performance outcomes with empirical validation?
- Does the study include robotic integration rather than focusing solely on computer vision without robotics?
- Is this a full research paper with substantial technical content rather than a conference abstract, editorial, opinion piece, or brief communication?

A large language model (LLM) was asked to extract data significant to this literature review, such as details about the ML approaches used, vision system, inspection application, performance metrics, manufacturing context, or system implementation. The search, followed by screening, identified 40 research papers examining ML-enhanced vision systems for robotic inspection across diverse manufacturing contexts.

The data extracted from each of the 40 publications was read and verified manually for consistency and correctness by the authors of this review, and 18 more papers were rejected based on the screening criteria. A typical criterion for which papers were rejected by the authors was when the topic focused on describing the use of ML-enhanced vision for robot manipulation, rather than describing its use in QC. Interestingly, none of the final 22 papers described the use of vision systems for inspection in welding or additive manufacturing technologies.

A second semantic search was performed using the Elicit search engine using the following query: “Machine learning-powered vision for robotic inspection in welding”. After a similar screening process and manual verification by the authors, an additional 18 research papers on vision inspection in robot welding were selected.

Finally, an additional 11 papers describing the use of ML-enhanced vision inspection in additive manufacturing were selected using the Google search engine.

**Figure 1:**
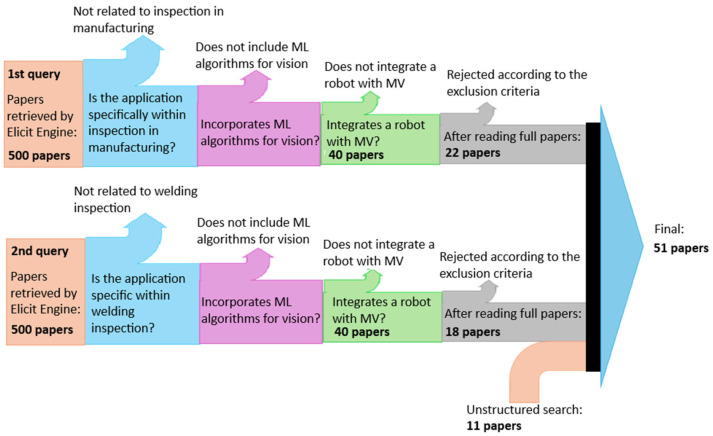
Sankey diagram illustrating the systematic literature selection process.

A Sankey diagram illustrating the systematic literature selection process is shown in Figure 1.

## 3. Results

### 3.1. Characteristics of the Selected Studies

The systematic review identified 51 studies examining state-of-the-art ML-enhanced robotic inspection in general manufacturing [22,23,24,25,26,27,28,29,30,31,32,33,34,35,36,37,38,39,40,41,42,43], in welding processes [44,45,46,47,48,49,50,51,52,53,54,55,56,57,58,59,60,61] and in additive manufacturing [62,63,64,65,66,67,68,69,70,71,72]. Table 1 presents the key characteristics of the studies addressing ML-enhanced inspection in general manufacturing processes.

Table 1 reveals substantial diversity in approaches across manufacturing sectors. Studies spanned across automotive, with 14% of the retrieved studies, aerospace (27%), assembly (10%), general manufacturing applications (27%), and other manufacturing sectors, including food processing, logistics/warehouse, molded part packaging, etc. (22%).

Most implementations remained at prototype or pilot scale—77%—with only 23% achieving full industrial deployment. This suggests that while the technology shows promise, barriers to widespread adoption persist.

Table 2 presents the key characteristics of the studies addressing ML-enhanced vision systems for robotic welding inspection.

The general manufacturing sector represented the largest application domain, accounting for approximately 33% of the retrieved studies, followed by the automotive industry, at 22%, and other specialized sectors, including aerospace, nuclear, pipeline construction, infrastructure, etc., accounting for 45% of the studies.

Laser welding was the most frequently studied processes, in 22% of studies, followed by Tungsten Inert Gas (TIG) welding (17%), arc welding (11%), and other welding processes, including fusion welding, resistance spot welding, or multilayer multi-pass welding, accounting for a total of 17% of the studies. A total of 33% of the studies did not specify the welding technology.

Production scale ranged from high-throughput mass production environments to the specialized inspection of critical components in aerospace manufacturing. Multiple studies emphasized real-time or inline inspection capabilities, addressing the need for immediate quality feedback in automated production lines.

Table 3 presents the key characteristics of the studies addressing ML-enhanced vision systems for inspection in additive manufacturing processes.

The studies spanned multiple AM processes: powder bed fusion (PBF) with both laser and electron beam was most common, with five studies, followed by directed energy deposition (DED), with two studies, material extrusion using filament fusion or liquid polymer extrusion using a syringe, with two studies, binder jetting, with one study, and other AM processes, with two studies.

Vision systems varied significantly in terms of the sensor type, positioning, and integration approach. The dominant sensor configuration was in situ camera monitoring, with positioning strategies including coaxial mounting aligned with the laser beam and off-axis mounting above the build chamber.

### 3.2. Machine Learning Technologies and Architectures

Both traditional ML and DL techniques are currently being used in vision inspection for manufacturing QC.

**Figure 2:**
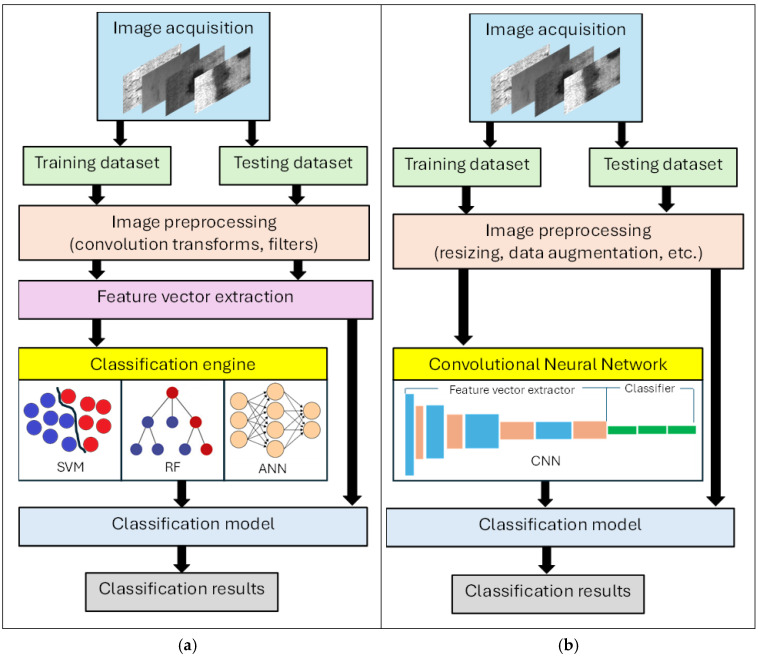
Manufacturing defects classification process (**a**) for traditional ML methods, and (**b**) for DL methods.

In traditional ML, vision detection and classification processes identify defects by comparing a set of their features, called feature vectors, to a set of features that are characteristic of classes of known defects. The classification process (Figure 2a) involves image preprocessing, feature vector extraction, feeding the feature vector to a classification engine, and evaluating the results. Training is achieved using a dataset of images of known defects and generally, the larger the dataset, the more accurate the classification process is. Image preprocessing is performed using classic MV convolution transforms to filter them, to eliminate insignificant features and keep only features that can be used for classification. The feature vectors can be extracted using algorithms such as the Histogram of Gradients (HOG), Histogram of Binary Patterns (HBP), etc. Feature vectors for classification based on color may include statistical functions of various color spaces, such as histograms, skewness, entropy, etc. Feature vectors for classification based on texture can be categorized as statistical (histograms, co-occurrence matrices, local binary descriptors, etc.), structural (edge features, morphological operations, etc.), model-based (fractal, random field, etc.), or transformer-based (spectral, wavelet, curvelet, etc.). Traditional machine learning classification engines include random forest (RF), Support Vector Machines (SVM), Nearest Neighbor, (1NN), K-Nearest Neighbor (KNN), Decision Tree (DT), single-layer Artificial Neural Networks (ANN), etc. Traditional ML works well with smaller, structured datasets, with fewer computational resources, and offers better interpretability when used with simpler models that are easier to understand and explain. Their disadvantage is that they require experts to select and transform the feature vectors from raw data, they may struggle with massive, complex or unstructured datasets, and they may not capture effectively intricate patterns in high-dimensional data.

Deep learning methods for defect detection and classification primarily use Convolutional Neural Networks (CNNs) to automatically learn features from raw pixels, moving beyond traditional methods like HOG or HBP. Key techniques involve hierarchical feature extraction in convolutional layers, training with large, labeled datasets (supervised learning), and using transfer learning (fine-tuning pre-trained models like AlexNet, VGG) for efficiency. Deep learning learns the feature vectors automatically from raw data; performance improves significantly with larger datasets, it excels with unstructured data and complex patterns, and it is more adaptable to complex, large-scale problems. Its disadvantages include that it needs a massive amount of labeled data to perform well, it requires significant computational power, it is hard to interpret how decisions are made, and it is more difficult to implement or tune.

The steps of defect detection and classification processes using CNNs are shown in Figure 2b.

**Figure 3:**
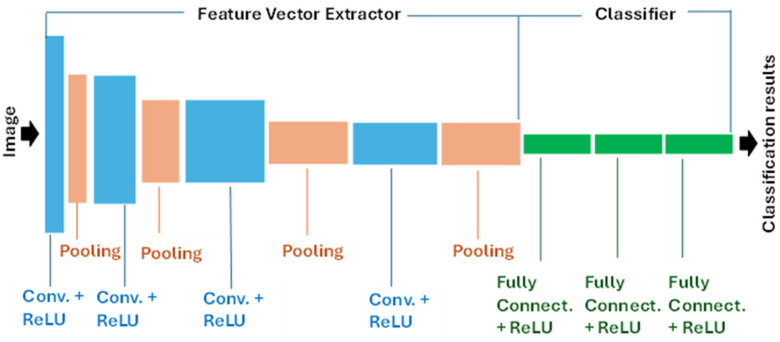
Basic structure of a CNN.

The basic structure of a CNN is shown in Figure 3. The CNN architecture contains an upstream feature vector extractor, also called the “backbone” or “body” of the network, and a downstream classifier, also called the “head” of the network. The backbone consists of convolutional layers which apply convolutional operations to input images using filters or kernels to detect features such as edges, textures, and more complex patterns. They also convert them to nonlinear values through their activation function, which is typically the Rectified Linear Unit (ReLU). Between convolutional layers, there are pooling layers, which downsample the input dimensions and reduce the number of parameters in the network. The fully connected layers are responsible for making predictions based on the features learned by the previous layers and may use ReLU or SoftMax activation functions.

CNNs used in manufacturing inspection can be categorized into four groups based on their application:

- Classic CNNs, which assigns a single class label to an entire image. Some representative networks used for this application are AlexNet, ResNet, or VGGNet.
- CNNs for defect detection and localization, which identify and locate defects with bounding boxes and assign individual class labels to each of them. Some representative networks used for defect detection and localization are R-CNN, faster R-CNN, or YOLO.
- CNNs for semantic segmentation, which assign a class label to each pixel in an image. They provide a holistic understanding of the image by segmenting it into meaningful semantic regions, without differentiating between individual object instances. Representative networks used for semantic segmentation are U-Net, FCN, DeepLab, PSP Net, or SegNet.
- CNNs for instance segmentation, which combine elements of defect detection and semantic segmentation. They identify and delineate individual defect instances within an image at a detailed pixel level and assign class labels to each identified defect. Representative networks used for instance segmentation are Mask R-CNN, Cascade Mask R-CNN, SOLO, or YOLACT.

All four categories of CNNs can be used for defect detection and classification, but CNNs for defect detection and localization and those for instance segmentation have the additional function of localizing the defects within the image.

**Figure 4:**
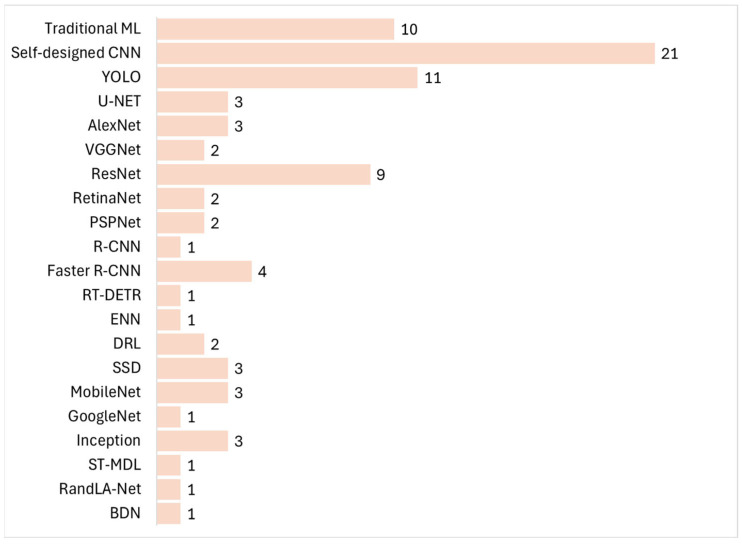
CNNs in vision inspection for manufacturing. The numbers indicate the frequency used. Traditional ML: refs. [25,36,47,49,51,56,59,61,62,63]. Self-designed CNN: refs. [24,27,30,34,36,37,39,44,45,46,49,53,55,56,60,62,63,64,70,71,72]. YOLO: refs. [22,28,29,31,33,43,50,54,57,58,69].U-Net: refs. [23,48,65]. AlexNet: refs. [54,63,67]. VGGNet: refs. [23,28,32]. ResNet: refs. [23,24,28,45,46,47,50,55,63]. RetinaNet: refs. [33,58]. PSPNet: refs. [26,38]. R-CNN: ref. [38]. Faster R-CNN: refs. [33,34,58,69]. RT-DETR: ref. [33]. ENN: ref. [41]. DRL: refs. [42,52]. SSD: refs. [35,58,69]. MobileNet: refs. [32,45,46]. GoogleNet: ref. [66]. Inception: refs. [45,47,55]. ST-MDL: ref. [49]. RandLA-Net: ref. [67], BDN [40].

The statistical analysis results of selected papers categorized by the type of CNN architecture used in manufacturing vision inspection are shown in Figure 4.

Self-designed CNNs represent the most frequently used network architectures in vision inspection, followed by YOLO, traditional ML, and ResNet. Self-designed CNNs represented combinations of CNN with the Long Short-Term Memory (LSTM) network, [39,44], a combination of CNN with the Gated Recurrent Unit (GRU) network [45,46], or were created using Keras, TensorFLow, and PyTorch libraries [24,71,72].

Traditional ML used classification engines such as RF [25,59,62], SVM [36,47,49,51,56,62], perceptron [51], Multi-Layer Perceptron (MLP) [36,62], DT [49], 1NN [61], KNN [49,56], or a combination of Bag of Words (BoW) and SVM [63].

### 3.3. Machine Learning Model Assessment

The ML model’s ability to identify and classify correctly defects were evaluated based on precision, recall, overall accuracy, F1 score, intersection over union, average precision, and mean average precision.

The precision for class *i*, $P_{i}$ represents the probability that a defect classified into class *i* does belong to class *i*, and is calculated as the ratio of the number of defects classified correctly into class *i* to the total number of defects classified into class *i*:

(1) $$P_{i}=\frac{TP_{i}}{TP_{i}+FP_{i}}$$

The recall for class *i*, $R_{i},$ is the probability that a defect is classified to the class to which it belongs, and is calculated as the ratio of the number of defects in class *i* classified correctly to the total number of defects that belong to class *i*:

(2) $$R_{i}=\frac{TP_{i}}{TP_{i}+FN_{i}}$$

The overall classifier accuracy, *OA* is defined as the total number of defects in the dataset classified correctly, $\underset{i}{\sum }TP_{i},$ divided by the total number of samples classified, *N*:

(3) $$OA=\frac{\sum_{i=1}^{M}TP_{i}}{N}$$

In Equations (1)–(3), $TP_{i}$ represents the true positives for class *i*, or the number of defects belonging to class *i* that were correctly predicted as belonging to that class, $FP_{i}$ represents false positives for class *i*, or number of defects belonging to other classes that were incorrectly predicted as belonging to class *i*, $FN_{i}$ represents false negatives for class *i*, or number of defects belonging to class *i* that were incorrectly predicted as belonging to other classes, *N* represents the total number of defects, and *M* represents the number of classes.

The *F*1*_i_* score is the harmonic mean of precision and recall, and provides a balanced assessment of a model’s performance while considering both false positives and false negatives:

(4) $$F1_{i}=2\times \frac{P_{i}\times R_{i}}{P_{i}+R_{i}}$$

The intersection over union—*IoU*—plays a fundamental role in evaluating the accuracy of defects’ localization and represents a measure that quantifies the overlap between a predicted bounding box and a ground truth bounding box:

(5) $$IoU=\frac{area\left(B_{predicted}\;\cap \;B_{actual}\right)}{area(B_{predicted}\;\cup \;B_{actual}}\times 100\%$$

The average precision for class *i*, *AP_i_*, represents the area under the precision–recall curve for class *i* and can be approximated using numerical integration.

The mean average precision, *mAP* is the mean of the *AP_i_* values across all classes in the dataset and is calculated as follows:

(6) $$mAP=\frac{1}{M}\sum_{i=1}^{M}AP_{i}$$

A more robust way to assess model performance is cross-validation, with the most commonly used version being k-fold cross-validation [73]. When different ML algorithms need to be compared, the most-used approach is nested cross-validation [74].

In industrial robotic inspection, metrics (1)–(6) are highly sensitive to dataset composition, controlled environments, and validation protocols. Defect-limited variability, class imbalance, controlled lighting, or lack of true production-scale testing represent potential sources of assessment bias. The reader must interpret the reported ML model assessment with caution.

Performance metrics were reported across multiple criteria, though not all studies provided comprehensive quantitative results. Detection and classification accuracy formed the primary metric, with processing speed, coverage ratios, and comparisons with baseline methods also frequently reported.

Multiple studies achieved exceptionally high accuracy rates, exceeding 95%. Variz et al. [30] achieved near-100% accuracy using a self-designed CNN used for vision quality control of Human–Machine Interface consoles. Ardic et al. [35] reported 99.9% accuracy using an R-CNN for engine part inspection after four months of operation. Terras et al. [33] demonstrated a detection and classification accuracy of 98%, successfully processing more than 600 items with high efficiency and low computational cost. Their results were matched by Shaloo et al. [31], who used YOLOv8 for assembly inspection.

The mid-90s accuracy range was commonly observed. Zhou et al. [38] reported 94.95% recall with 92.35% precision for mesh screen inspection. Rajesh et al. [25] achieved 95% accuracy and 94% recall using an RF classification engine in vision inspection of gears. Yazid et al. [43] demonstrated 96% detection accuracy using YOLOv5, while Hussain et al. [32] obtained a 92.7% mean average precision for pallet racking inspection using VGG16 network.

Lower accuracy ranges were observed in more challenging applications. Mueller et al. [37] reported 86% accuracy for online rivet classification using sensor data, improving to 97% with image-based classification using a self-designed CNN. Lee et al. [41] achieved 83.33% accuracy using an ENN classifier, correctly predicting five of six datasets in hole quality assessment.

When specific algorithm comparisons were provided, performance differences emerged. For engine part inspection, Ardiç et al. [35] found that Faster R-CNN achieved 0.994 average precision versus 0.955 for SSD. Kirda et al. [28] compared three algorithms for metal edge detection: YOLOv5 achieved 0.957 mean average precision, outperforming VGG16 at 0.942 and ResNet at 0.854. The superiority of ensemble methods (Knaak et al. [45] 99.5% F1 score; Knaak et al. [46] 95.2% F1 score) versus single-model approaches stems from their ability to combine complementary error patterns. Spatiotemporal CNN-GRU architecture captured dynamic welding process features that pure spatial CNNs missed. Similarly, Fernandez et al. [44] found a superior performance of the spatiotemporal CNN-LSTM architecture compared to pure spatial CNN (0.95 vs. 0.94 recall).

#### Context-Specific Performance Patterns

Performance outcomes cluster distinctly by application complexity and environmental conditions. Studies achieving the highest accuracy (>98%) predominantly addressed well-defined defect categories in controlled environments. O. Ardiç et al.’s [35] 99.9% accuracy for engine parts and N. Terras et al.’s [33] 98% for food products occurred in assembly line settings with consistent object presentation and minimal environmental variability.

In contrast, applications in unstructured or dynamic environments showed systematically lower performance. In the study by R. Mueller et al. [37], aircraft riveting inspection achieved only 86% accuracy when relying on real-time sensor data, improving to 97% with post-process image analysis, suggesting that temporal constraints in collaborative human–robot scenarios compromise detection reliability.

The L. Variz et al. [30] study illustrates performance variability within a single system: while console classification and button defect detection approached 100% accuracy, face recognition exceeded only 50%. This dramatic difference reflects the fundamental distinction between inspecting manufactured components with consistent specifications versus recognizing variable human features, suggesting that performance claims require careful scoping to specific subtasks rather than system-level averages.

The apparent contradiction between traditional ML outperforming DL in specific cases (e.g., in the study by S. Zhang et al. [56], KNN achieved 98% accuracy in 33 ms versus the slower CNN performance) resolves when considering feature space dimensionality. KNN excelled when discriminative features were already well-understood and extracted using Gabor transforms and texture analysis, whereas DL demonstrated advantages when feature engineering was not feasible.

### 3.4. Machine Learning Architecture Trade-Offs

The prevalence of YOLO variants across studies reflects not superior fundamental performance but rather a favorable balance of speed, accuracy, and simplicity of deployment in industrial applications. Direct algorithmic comparisons reveal nuanced trade-offs rather than clear winners. O Ardiç et al. [35] found that Faster R-CNN achieved higher average precision (0.994) than SSD (0.955) for engine inspection, yet SSD’s faster inference might prove preferable in high-throughput scenarios despite lower accuracy. A.W. Kirda et al.’s [28] comparison showed YOLOv5 (0.957 MAP) outperforming VGG16 (0.942) and ResNet (0.854), but the 0.015 advantage over VGG16 may not justify switching in systems already using the latter.

## 4. Future Directions

Future directions in ML-powered vision for manufacturing QC are driven by the demand to achieve higher accuracy, robustness, and reliability.

### 4.1. Use of Synthetic Training Images

Limited training data has been revealed as a primary constraint on performance and deployment of ML models, explaining why many high-performing vision systems remain at prototype scale. Transfer learning can mitigate for limited datasets but does not eliminate data requirements for production reliability.

Synthetic training images can boost the reliability of machine learning vision by providing vast, perfectly labeled, diverse data, especially for rare edge cases that are hard to obtain in real life. Automated synthetic data generation enables rapid scaling, reduces bias, and protects privacy while delivering high-quality annotation. In computer vision, synthetic data generation uses advanced techniques like generative adversarial networks (GANs) and variational autoencoders (VAEs). These models learn patterns from real datasets and then produce new, artificial examples.

### 4.2. Use of Federated Machine Learning

A second direction that has the potential to increase the size of training datasets while improving data privacy and security in manufacturing is through federated learning. Federated Machine Learning is a decentralized AI training method that builds a shared model from data on many devices without moving the raw data, keeping sensitive information private. Instead of sending data to a central server, the model travels to the data, learns locally, and sends back only aggregated updates such as model parameters or gradients to improve the main model.

### 4.3. Ensemble Learning

Ensemble learning combines multiple individual models to create a single, more powerful model, improving prediction accuracy, robustness, and generalization by leveraging collective “wisdom” over a single model. This review has already shown that hybrid spatiotemporal models such as CNN-GRU or CNN-LSTM outperform the pure spatial CNN models.

### 4.4. Self-Supervised Learning

Training on image datasets requires manually adding labels to objects in images, a time-consuming process called annotation. An emerging direction is Self-Supervised Learning (SSL), a type of ML where models learn to generate labels from the input data itself, eliminating the need for manually labeled images. SSL models for industrial vision train on unlabeled image/video data to learn features for tasks like defect detection, object recognition, and anomaly localization, reducing reliance on costly annotations. They enable powerful Vision Foundation Models like DINOv3 to adapt better to specialized industrial environments where labeled data is scarce. The key benefits of SSL models include lower costs, faster training, and improved performance on downstream tasks.

### 4.5. Visual–Language Models for Explainability

Visual–Language Models (VLMs) enhance explainability by generating human-readable descriptions, identifying important visual features, and providing step-by-step reasoning for complex tasks, thus bridging the gap between opaque AI decisions and user understanding. VLMs can generate natural-language descriptions of visual content, moving beyond simple labels to explain why objects are recognized. They can highlight specific image regions or features such as pixels or objects that are most influential in their decision-making process. VLMs use techniques such as Chain-of-Thought prompting to outline their reasoning steps, making complex logical paths such as puzzles and medical diagnosis understandable. Their benefit is in making AI decisions understandable to non-experts by translating complex computations into simple language, and uncovering false correlations or biases by revealing the model’s thinking process, thus increasing user confidence.

### 4.6. Physics-Informed Machine Learning

Physics-Informed Machine Learning (PIML) integrates known physical laws such as the conservation of energy or fluid dynamics equations directly into machine learning models, creating more accurate, data-efficient, and physically consistent AI systems. They become especially useful when data is scarce, or the underlying physics is complex. They work by adding physical laws, often expressed by partial differential equations, to the ML’s loss function, thus penalizing predictions that violate these laws. By embedding physical knowledge, models need significantly fewer training samples to generalize well, overcoming the data-scarcity issues common in science and engineering.

## 5. Conclusions

This review provides insight into the current landscape and state-of-the-art practices in smart manufacturing ML-powered robotic vision inspection.

More than 50 studies spanning across the automotive, aerospace, assembly, and general manufacturing sectors demonstrate that ML-powered vision is a technical viability for robotic inspection in manufacturing.

The accuracy of defect detection and classification frequently exceeds 95%, with some vision systems achieving 98–100% accuracy in controlled environments.

The vision systems use predominantly self-designed convolutional neural network (CNN) architectures, YOLO variants, or traditional ML vision models.

However, 77% of implementations remain at prototype or pilot scale, revealing systematic deployment barriers.

## Figures and Tables

**Table 1:** Key characteristics of the studies addressing ML-enhanced inspection in general manufacturing.

Study	Industry Sector	ML Technique	Vision System	Robot	Inspection Application	Deployment Scale
A. Villalonga et al. [22]	General manufacturing	YOLOv10	Mako G192	UR5e	QC	Pilot line
A. Rosell et al., 2023 [23]	Aerospace	U-Net, VGG16, ResNet50	Not specified	Unspecified	Aerospace engine components	Deployed system
D. Kim et al., 2023 [24]	Assembly manufacturing	Self-designed CNN, ResNet50	Dual cameras	Unspecified	Peg-in-hole assembly quality	Pilot/prototype
P. J. Rajesh et al., 2024 [25]	Automotive (gears)	RF	Unspecified	Unspecified	Gear teeth defects (cracks, chips, wear)	Pilot/ prototype
J. Chen et al., 2023 [26]	Printing (ink bags)	PSPNet	Single and multi-camera systems	Unspecified	Air bubble volume in ink bags	Pilot/full deployment
J. N. Karigiannis et al., 2021 [27]	Aerospace	Self-designed CNN	Unspecified	Fanuc LR-MATE 200iD	Fluorescent penetrant inspection	Proof-of concept
A. Kirda et al., 2025 [28]	General manufacturing	YOLOv5, VGG16, ResNet	Unspecified	Mitsubishi	Metal edge detection	Proof-of-concept
Liwei Zeng et al., 2025 [29]	Industrial inspection	YOLOv5	Unspecified	Unspecified	Detect objects in robot workspace	Prototype/pilot
L. Variz et al., 2019 [30]	HMI console manufacturing	Self-designed CNN	Mako G125b	UR3e	Button condition, LCD display defects	Prototype/pilot
M. Shaloo et al., 2024 [31]	Parts assembly	YOLOv8	Unspecified	Mitsubishi	Assembly correctness	Prototype/pilot
M. Hussain et al., 2022 [32]	Logistics/warehouse	MobileNet	Smartphone camera		Pallet racking damage	Prototype/pilot
N. Terras et al., 2025 [33]	Food products	RetinaNet, RT-DETR, Faster RCNN, YOLO	Unspecified	UR3e	Food sorting and quality	Pilot/full deployment
N. Raj et al., 2024 [34]	General manufacturing	Self-designed CNN	Unspecified	Unspecified	Sheet-metal defects (scratches, dimensional deviations)	Prototype/pilot
O. Ardiç et al., 2024 [35]	Automotive	Faster R-CNN, SSD	Unspecified	Fanuc CR-15ia	Engine part defects	Full deployment
P. Bauer et al., 2022 [36]	Automotive	Self-designed CNN, SVM, MLP	ZEISS COMET 3D Sensor, Canon DSLR	Fanuc M-20ia	Sheet metal reference markers	Prototype/pilot
R. Mueller et al., 2019 [37]	Aerospace	Self-designed CNN,	Laser line Sensor + RGB camera	Unspecified	Rivet quality	Prototype/pilot
S. Zhou et al., 2025 [38]	Molded pulp packaging	R-CNN, PSPNet	USB camera	UR3e	Clogged pores in mesh screens	Pilot/full deployment
S. Martelli et al., 2018 [39]	Aerospace	Self-designed CNN, CNN + LSTM	Microcamera in endoscope	ABB IRB1600	Gearbox residuals	Prototype/pilot
S. Deshpande et al., 2023 [40]	Aerospace (sealant)	BDN	Unspecified	KUKA KR Agilus	Glue dot quality	Prototype
S. K. H. Lee et al., 2025 [41]	Aerospace	ENN	Unspecified	KUKA KR210	Hole quality in composites	Prototype/pilot
W. Tang et al., 2023 [42]	General manufacturing	DRL	RGB camera	Unspecified	Cracks on metallic surfaces	Prototype/pilot
Y. Yazid et al., 2023 [43]	General manufacturing	YOLOv5	RGB-D camera	UR5	Metal part defects on conveyor	Prototype/pilot

**Table 2:** Key characteristics of the studies adresssing ML-enhanced vision systems for robotic welding inspection.

Study	Primary Application	ML Technique	Robot	Welding Process	Industrial Context
A. Fernández et al., 2020 [44]	Online monitoring	Self-designed CNN, CNN + LSTM	ABB	Arc welding	Unspecified
C. Knaak et al., 2021 [45]	Real-time defect detection	Self-designed CNN, CNN + GRU, ResNet50, MobilNetV2, InceptionV3	Unspecified	Laser welding	Automotive/ aerospace
C. Knaak et al., 2021a [46]	Fault detection	Self-designed CNN, CNN + GRU, ResNet50, MobilNetV2, InceptionV3	Unspecified	Laser welding	Manufacturing
C. Xia et al., 2020 [47]	State recognition	ResNet, SVM	Unspecified	Keyhole TIG	Manufacturing
D.D. Kumar et al., 2023 [48]	Porosity detection	ST-MDL, U-Net	Unspecified	Unspecified	Infrastructure
D. Buongiorno et al., 2022 [49]	Defect classification	Self-designed CNN, DT, SVM, KNN	Comau NJ220	Laser welding	Automotive (EV batteries)
H. Li et al., 2023 [50]	Defect detection	YOLOv5, ResNet50	Unspecified	Arc welding	Automotive bracket production
M. Yemelyanova et al., 2024 [51]	Surface defect recognition	Perceptron, SVM	Unspecified	TIG welding	Pipe production
Markus Schmitz et al., 2020 [52]	Quality evaluation	DRL	Unspecified	Laser welding	Unspecified
N. Cherkasov et al., 2023 [53]	Surface defect detection	Self-designed CNN	Fanuc ARC Mate	Unspecified	Steel structures
O. Kartashov et al., 2022 [54]	Pipeline weld inspection	YOLOv5	Unspecified	Fusion welding	Pipeline installation
S. Kajan et al., 2024 [55]	Quality inspection	Self-designed CNN, AlexNet, ResNet18, Inception-v3	Fanuc CRX-25ia	Unspecified	Unspecified
S. Zhang et al., 2022 [56]	Real time defect recognition	Self-designed CNN, SVM, KNN	Unspecified	TIG welding	Automation
Van-Doi Truong et al., 2025 [57]	Multi-pass monitoring	YOLOv10	Unspecified	Multi layer multi pass welding	Nuclear pressure vessels
W. Dai et al., 2021 [58]	Spot weld inspection	YOLOv3, SSD, Faster R-CNN, RetinaNet	Fanuc	Resistance spot welding	Automotive
X. Dong et al., 2020 [59]	Defect inspection	RF	Unspecified	Unspecified	Aerospace
Y. J. Cruz et al., 2020 [60]	Pre/post-weld inspection	Self-designed CNN	Unspecified	Unspecified	LPG pressure vessels
Yun Shi et al., 2023 [61]	Surface defect detection	1NN	Unspecified	Unspecified	Industrial manufacturing

**Table 3:** Key characteristics of the studies adresssing ML-enhanced vision systems for inspection in additive manufacturing (AM) processes.

Study	AM Process	Materials	ML Approach	Sensor Types
A. Gaikwad et al., 2022 [62]	Laser Powder Bed Fusion	Metals (inferred)	Self-designed CNN, SVM, MLP, RF, KNN	Two co-axial high-speed cameras, thermal imaging
A. Rossi et al., 2021 [63]	Fused Filament	Unspecified	Self-designed CNN, AlexNet, ResNet50, BoW + SVM	Digital camera
B. Zhang et al., 2019 [64]	Metal AM	CoCrMo	Self-designed CNN	Unspecified
D. Cannizzaro et al., 2022 [65]	Powder Bed Fusion	Metals	U-Net	Off-axis camera
E. Tsintavi et al., 2024 [66]	Material Extrusion using syringe	Orodispersible films with Warfarin	GoogleNet	Camera (inferred)
F. Kaji et al., 2022 [67]	Laser Direct Energy Deposition via powder feeding	Metals	RandLA-Net	Laser line scanner
H. Elwarfalli et al., 2019 [68]	Laser Powder Bed Fusion (Selective Laser Melting)	Metals	AlexNet	IR tomography
L. Lu et al., 2023 [69]	Robot-based Composite Fiber-Reinforced Polymer AM	Composite Fiber-Reinforced Polymer	Faster R-CNN, SSD, YOLOv4	Unspecified
L. Scime et al., 2020 [70]	Powder Bed Fusion (laser fusion, binder jetting, and electron beam fusion)	Unspecified	Self-designed CNN	Unspecified
V. Klamert et al., 2023 [67]	Laser Powder Bed Fusion	Polyamide PA2200	Self-designed CNN	Low-cost RGB Camera (Raspberry Pi)
Z. Chen et al., 2025 [68]	Laser Direct Energy Deposition	Metals	Self-designed CNN	Unspecified

## Data Availability

No new data were created or analyzed in this study. Data sharing is not applicable to this article.

## Abbreviations

The following abbreviations are used in this manuscript:

AI	Artificial Intelligence
AM	Additive Manufacturing
BDN	Bayesian Decision Networks
BoW	Bag of Words
CNN	Convolutional Neural Network
DL	Deep Learning
DRL	Deep Reinforcement Learning
DT	Decision Tree
ENN	Ensemble Neural Network
F1_i_	F1 score for class i
GAN	Generative Adversarial Network
GRU	Gated Recurrent Unit
HBP	Histogram of Binary Patterns
HOG	Histogram of Gradients
IoU	Intersection over union
KNN	K-Nearest Neighbor
LLM	Large Language Model
LSTM	Long Short-Term Memory
mAP	Mean average precision
ML	Machine Learning
MLP	Multi-Layer Perceptron
NN	Neural Network
1NN	Nearest Neighbor
OA	Overall classifier accuracy
P_i_	Precision for class i
QC	Quality Control
R_i_	Recall for class i
RF	Random Forest
RT-DETR	Real-Time Detection Transformer
ST-MDL	Semi-supervised Transfer Learning based Multi-Domain learning
SSD	Single Shot Detector
SVM	Support Vector Machine
VAE	Variational Autoencoder
